# Developing a National Research Agenda to Support Healthy Food Retail

**DOI:** 10.3390/ijerph17218141

**Published:** 2020-11-04

**Authors:** Amelie A. Hecht, Megan M. Lott, Kirsten Arm, Mary T. Story, Emily Snyder, Margo G. Wootan, Alyssa J. Moran

**Affiliations:** 1 Department of Health Policy and Management, Johns Hopkins Bloomberg School of Public Health, Baltimore, MD 21205, USA; AMoran10@jhu.edu; 2 Healthy Eating Research, Duke Global Health Institute, Duke University, Durham, NC 27708, USA; megan.lott@duke.edu (M.M.L.); kirsten.arm@duke.edu (K.A.); mary.story@duke.edu (M.T.S.); 3 Center for Science in the Public Interest, Washington, DC 20005, USA; esnyder@cspinet.org (E.S.); mwootan@mxgstrategies.com (M.G.W.)

**Keywords:** food and beverage, grocery retail, supermarket, marketing, policy, research agenda, healthy food retail, food environment

## Abstract

The food retail environment is an important driver of dietary choices. This article presents a national agenda for research in food retail, with the goal of identifying policies and corporate practices that effectively promote healthy food and beverage purchases and decrease unhealthy purchases. The research agenda was developed through a multi-step process that included (1) convening a scientific advisory committee; (2) commissioned research; (3) in-person expert convening; (4) thematic analysis of meeting notes and refining research questions; (5) follow-up survey of convening participants; and (6) refining the final research agenda. Public health researchers, advocates, food and beverage retailers, and funders participated in the agenda setting process. A total of 37 research questions grouped into ten priority areas emerged. Five priority areas focus on understanding the current food retail environment and consumer behavior and five focus on assessing implementation and effectiveness of interventions and policies to attain healthier retail. Priority topics include how frequency, duration, and impact of retailer promotion practices differ by community characteristics and how to leverage federal nutrition assistance programs to support healthy eating. To improve feasibility, researchers should explore partnerships with retailers and advocacy groups, identify novel data sources, and use a variety of study designs. This agenda can serve as a guide for researchers, food retailers, funders, government agencies, and advocacy organizations.

## 1. Introduction

The food retail environment is an important driver of dietary choices in the U.S. Components of the food retail environment, including access to food retail, availability and price of healthy products in stores, and presence of in-store marketing, all play a role in shaping dietary patterns [1,2]. Food and beverage manufacturers spend billions of dollars annually to ensure retailers stock, prominently place, and promote their products [3]. Unhealthy products are promoted more often than healthy products, and evidence suggests that promotion of unhealthy products shapes consumer purchasing more than promotion of healthy products [4,5].

Current dietary patterns, which, compared to the *2015–2020 Dietary Guidelines for Americans,* are low in fruits and vegetables, whole grains, and lean protein, and high in sodium, added sugars, and saturated fat, put many Americans at elevated risk of chronic health conditions, including obesity and diabetes [6,7]. Low-income and racial/ethnic communities, who experience greater prevalence of diet-related chronic diseases, may also be more likely to be targeted by marketing of unhealthy foods and beverages [8,9,10]. For example, in-store marketing of unhealthy beverages has been shown to increase at the time of month when Supplemental Nutrition Assistance Program (SNAP) benefits are distributed, particularly in neighborhoods with high SNAP participation [10].

As national attention toward health disparities and diet-related chronic diseases has increased in recent years, researchers, advocates, and policymakers have recognized the need to improve the food retail environment. In 2010, the Robert Wood Johnson Foundation (RWJF) and The Food Trust convened researchers, public health advocates, food retailers, manufacturers, and marketing professionals to discuss strategies to promote healthy retail, with a particular focus on children in low-income communities. The report that followed, *Harnessing the Power of Supermarkets to Help Reverse Childhood Obesity*, recommended marketing tactics to promote healthier purchases that jointly benefited consumers, retailers, and manufacturers [11]. In the intervening years, progress has been made toward identifying retail practices that undermine healthy eating and designing interventions that promote healthy eating in the retail food environment. At the same time, the retail food landscape has evolved: grocery store chains have consolidated, dollar stores have gained market share, and some consumers have shifted their purchases online. Research to fill remaining and emerging gaps in the food retail literature is needed.

This article outlines a national research agenda to support healthy food retail developed by Healthy Eating Research (HER; a national program of RWJF), the Center for Science in the Public Interest (CSPI), The Food Trust, and other researchers. This is the first national research agenda focused on healthy food retail. Research agendas have been developed to guide work on a variety of other public health topics [12,13]. Agenda-setting helps to identify important gaps in knowledge and to build consensus and support to fill those gaps among funders, advocates, and researchers. This agenda describes key areas for research to better understand current food retail practices and consumer behaviors and potential retail strategies to promote healthy eating while addressing racial and income disparities in diet quality and related disease. Research in these domains can inform policy strategies and corporate practices to improve the food retail environment and promote health equity. This article describes the collaborative and iterative methods used to develop the research agenda and the results generated at each step of the process. It then presents a final set of research questions in a comprehensive research agenda, key considerations for how to conduct that research, and ways in which the research agenda can be used to advance the field and public health.

## 2. Methods

**Figure 1:**
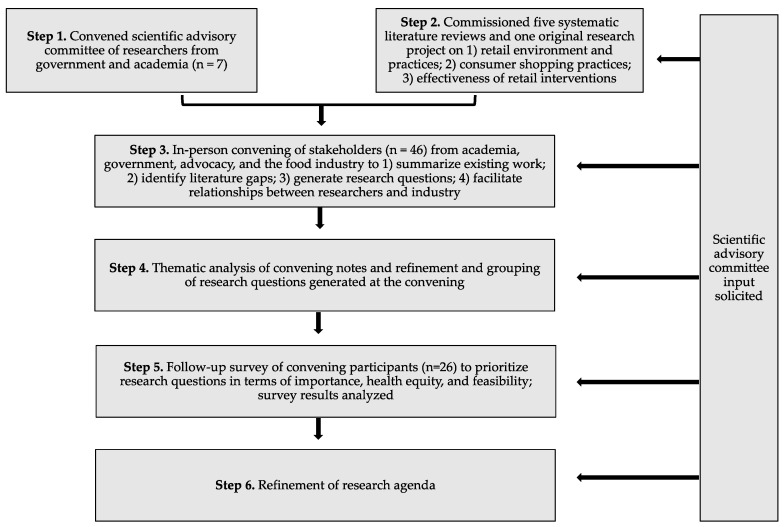
Flow chart depicting the process of developing the national healthy retail research agenda.

The research agenda was developed through an iterative process between October 2019 and July 2020 that included the following steps: (1) convening a scientific advisory committee; (2) commissioning five systematic literature reviews and one original research project on food retail practices and interventions; (3) in-person convening of expert stakeholders; (4) thematic analysis of meeting notes and refining research questions; (5) follow-up survey of convening participants; and (6) developing the final research agenda (Figure 1). The scientific advisory committee provided input at each stage of the process. This agenda-setting process was based on methods used by Duffy et al. [12].

The research agenda was developed with an emphasis on health equity and the demographic groups that are at highest risk for poor health, especially nutrition and weight-related health disparities. These priority populations, identified by HER, include Non-Hispanic Black, Hispanic/Latinx, Asian American, Native Hawaiian/Pacific Islander, American Indian/Alaska Native, and rural children and their families [14].

### 2.1. Convening a Scientific Advisory Committee

A scientific advisory committee was formed and included seven researchers from government, academia, and nonprofit organizations, representing a variety of substantive areas related to psychology, nutrition, health behavior, anthropology, and public policy. The committee was selected based on prior work in the field, leadership in related working groups and professional organizations, and experience working with HER’s priority populations. The committee provided input on topics for commissioned research, the in-person convening agenda and guest list, and content of the follow-up survey and final research agenda. Committee members also took notes and guided small group discussions at the in-person convening. 

### 2.2. Commissioned Research

Five literature reviews and one original research project were commissioned for the in-person convening and were conducted by experts in the field. (Five of these papers are published jointly with this special issue.) These works aimed to provide an overview of previous research on key topics and guide convening discussion. Commissioned papers were organized into three themes: (1) retailer and manufacturer marketing practices, (2) consumer food purchasing trends by race/ethnicity, socioeconomic status, and geographic location, and (3) effectiveness of government- and researcher-led retail interventions to increase healthy food access and purchases. The original research paper used Nielsen Homescan Consumer Panel data from 2008–2018 to assess how packaged food purchases differ by store type and consumer demographics (urban vs. rural, high vs. low income).

### 2.3. In-Person Convening of Expert Stakeholders

The goals of the in-person convening were to (1) summarize previous research on healthy food retail, (2) identify gaps in the literature, (3) generate and prioritize questions for future research, (4) highlight best practices for research collaboration with the food industry, and (5) facilitate relationships between retailers and researchers to implement and evaluate healthy retail interventions. The full-day event was held in Washington, DC on 29 January 2020 and was organized by staff from HER, CSPI, and The Food Trust and the scientific advisory committee. Forty-six expert stakeholders from academia, government, advocacy, and the food industry participated. 

In advance of the meeting, participants were asked to read six brief reports with the preliminary findings from the five commissioned systematic reviews and one original research project. At the convening, academic researchers presented key findings from each of the commissioned projects. Presentations were grouped according to the three themes discussed in Section 2.2 (two presentations per theme). After each pair of presentations, scientific advisory committee members facilitated small group breakout discussions. In breakout groups, participants discussed findings from the presentations and research gaps related to the theme, including understudied populations. Participants were asked to brainstorm new research methods, data sources, and study designs to facilitate future evaluation. 

Meeting organizers also facilitated a large group discussion during which participants were asked where they would recommend directing intervention research over the next ten years to have the greatest impact on population health and equity. Subsequently, a panel of industry representatives discussed best practices for researchers seeking to partner with retailers and food manufacturers on healthy retail research. Finally, in small groups, participants were asked prioritize research questions identified throughout the day that would help fill knowledge gaps.

After each small and large group discussion, participants were asked to write research questions that emerged on sticky notes. Sticky notes were placed on walls throughout the meeting room according to the theme. At the end of the convening, participants were asked to walk around the room and place dots next to the research questions they thought were most important for advancing health equity. 

### 2.4. Thematic Analysis of Notes and Refinement of Research Questions

Notes taken by the scientific advisory committee at the convening and sticky notes generated by convening participants were thematically analyzed and grouped by three authors collaboratively (A.A.H., M.G.W., A.J.M.). The list of research questions was collated and refined by deleting duplicate questions, questions that were too vague or specific, and questions outside the scope of the research agenda. Cross-cutting considerations related to study design, setting, data sources, and partnerships raised during group discussions were also refined.

### 2.5. Follow-Up Participant Survey

An online follow-up survey was sent via email to convening participants in May 2020. The survey was developed by the authors with feedback from the scientific advisory committee. The survey was first entered into Qualtrics and tested for functionality and length. Respondents were asked to complete the survey within two weeks, during which time two reminder emails were sent.

A total of 40 research questions generated at the in-person convening were included in the follow-up survey. Survey respondents were asked to rank each research question on a scale from 1 (lowest) to 5 (highest) in terms of feasibility, equity, and importance (defined in Table 1). For each research question, average scores for each domain and composite scores were calculated using Microsoft Excel. Research questions that received low composite scores (<3) or low scores in all three domains (<3.5) were removed. This allowed research questions that received low scores in one domain but high scores in one or both of the remaining domains to be preserved (for example, a question that received a score of 2.0 for feasibility but a score of 3.7 for importance and 3.5 for health equity would be preserved). Respondents were asked to list any missing research questions. Finally, respondents were provided a list of data sources for healthy retail research identified at the convening and provided an opportunity to list additional data sources.

### 2.6. Developing a Final Research Agenda

The final research agenda was developed based on findings from steps 2–5 (see Figure 1) and with critical input from the scientific advisory committee and select members of the Healthy Food Retail Working Group, which is supported by HER and the Centers for Disease Control and Prevention’s (CDC) Nutrition and Obesity Policy Research and Evaluation Network (NOPREN). The final research questions (selected based on follow-up survey results) and the cross-cutting considerations for research were grouped into key themes. 

## 3. Results

### 3.1. Commissioned Research Findings

Key findings from the commissioned research papers, including research gaps, are discussed briefly here; five of the commissioned papers are also published in this special issue. 

Two commissioned systematic reviews focused on retailer and manufacturer marketing practices. The first identified four key strategies that food and beverage manufacturers use to influence retailer marketing practices, but called for further research to understand the role that financial incentives from manufacturers play in shaping the retail environment, including analyses using proprietary data from retailers and manufacturers [15]. The review also found evidence that retailer marketing strategies, including price discounts and prominent store placement, are associated with increased product sales, but concluded that other in-store promotional strategies, such as signs and displays, are understudied. A second commissioned paper assessed marketing-mix and choice-architecture (MMCA) strategies used to promote and sell sugar-sweetened beverages (SSBs) in U.S. food stores and found that SSBs were widely available and price reductions and promotions were used often to boost sales. The authors found that targeted MMCA strategies may be used to influence SSB purchases among at-risk consumers on the basis of income or race/ethnicity, for example, and that MMCA strategies may vary by retail format. They noted that most studies were not designed to capture such differences, representing a need for future investigation to inform practice and policy approaches to mitigate health disparities [16]. 

Two additional commissioned papers focused on differences in consumer shopping patterns by race/ethnicity, socioeconomic status, and geographic location (urban vs. rural). In one systematic review, the authors called for more research that examines how these three factors intersect to influence U.S. consumer food purchasing [17]. In particular, they found a small proportion of included studies examined purchasing at the intersection of two factors (race/ethnicity and socioeconomic status), and no studies examined purchasing at the intersection of all three factors or assessed geographic differences in purchasing. The other paper, an original research project using household packaged food purchase panel data from 2008–2018, identified heterogeneity in the type and nutritional quality of packaged foods and beverages purchased by urban versus rural households and low- versus high-income households in different retail formats [18]. The authors called for research to examine why these differences exist—for example, why rural households tend to buy more packaged foods from mass merchandisers and dollar stores, which offer foods of poorer nutritional quality.

The final two commissioned systematic reviews examined the impact of retail interventions on consumers and retailers. One review synthesized 148 evaluations of governmental policies designed to increase healthy food purchases in supermarkets and found that sweetened beverage taxes, revisions to the Special Supplemental Nutrition Program for Women, Infants and Children (WIC) food packages, and financial incentives for fruits and vegetables were associated with improvements in dietary behaviors [19]. Providing financial incentives to supermarkets to open in underserved areas and increases in SNAP benefits were not associated with changes in diet quality but may improve food security. The authors called for more research to understand the effects of calorie labeling in supermarkets and online SNAP purchasing on consumer purchasing and consumption. The second paper reviewed 64 in-store marketing studies conducted between 2009–2019 and found that the majority of interventions identified at least one positive effect related to healthier food purchasing, consumption, or sales. Promotion was the most commonly studied marketing strategy for single-component interventions, while changing promotion, placement, and product together were the most common for multi-component interventions. The quality of research, however, precluded definitive conclusions, as fewer than 36% of studies used experimental designs. The review called for more research to understand what combinations of strategies work best by product category and retail format [20]. 

### 3.2. In-Person Convening Findings

**Figure 2:**
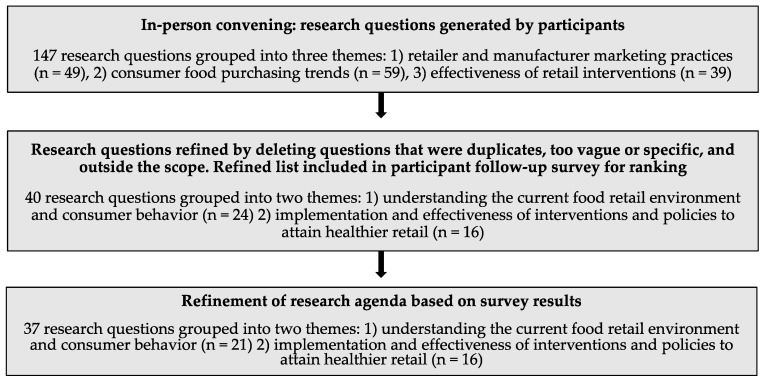
Flow chart depicting how research questions were generated and refined through the agenda-setting process.

Research questions generated at the meeting (n = 147) were initially grouped according to the three meeting agenda themes (retailer and manufacturer marketing practices; consumer food purchasing trends; and effectiveness of retail interventions). (Figure 2) Forty-nine questions fell under the retailer and manufacturer marketing practices theme, 59 under the consumer food purchasing trends theme, and 39 under the effectiveness of retail interventions. These questions were refined and reorganized prior to inclusion in the follow-up survey. Two themes—retailer and manufacturer marketing practices and consumer food purchasing trends—were condensed due to overlap between research questions in these categories. In total, 40 questions representing two themes were included in the follow-up survey. 

### 3.3. Follow-Up Survey Findings

Twenty-six convening attendees completed the follow-up survey (response rate 57%). Three research questions were eliminated due to low scores: one question earned a low composite score (<3), and two questions earned low scores across all three domains (<3.5) (Table A1).

**Figure 3:**
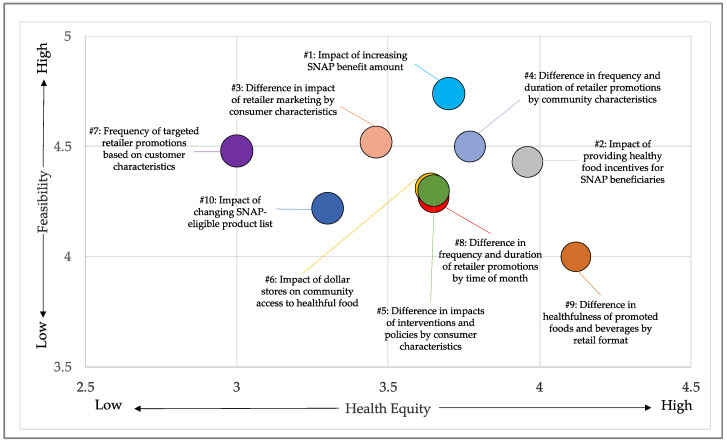
Research questions with the 10 highest composite scores from the follow-up survey. Numbers listed before questions represent ranking from 1–10 by composite score. Research questions were ranked on a 5-point Likert scale in terms of feasibility (y-axis) and health equity (x-axis). Ratings for importance are not displayed due to low variation (3.8–4.5) among the top ten questions. SNAP is the Supplemental Nutrition Assistance Program.

Research questions that received the highest composite scores focused on describing how frequency, duration, and impact of retailer promotion practices differed by community characteristics and how to leverage SNAP benefits to support healthy eating behaviors. (Figure 3) Research questions that received the highest scores for importance and equity focused on (1) evaluating the impact of retailer marketing practices on consumer health, (2) understanding the optimal retail design to promote healthy and reduce unhealthy purchases, and (3) evaluating the impact of healthy retail policies to address the social determinants of health. These questions, however, received lower scores for feasibility. Research questions that received the highest scores for feasibility focused on describing the current retail environment, including assessing the healthfulness of products currently available and promoted in stores, and describing the factors that influence consumer decision-making.

In the open-ended portion of the survey, several participants suggested additional research questions related to the COVID-19 pandemic. The research questions that participants were asked to rank were generated at the January convening, before widespread awareness of COVID-19 pandemic in the U.S., but the survey was conducted in May during the pandemic. A few participants indicated an interest in evaluating how COVID-19, generally, and the U.S. Department of Agriculture (USDA) waivers during the pandemic for SNAP and WIC statutory and regulatory requirements, specifically, affected food supply, retailer marketing, and consumer purchasing. Another participant called for research on how expansion of the SNAP Online Purchasing Pilot Program (a federal program to test the feasibility and impact of allowing online food retailers to accept SNAP benefits [21]) affects small and independent grocers. 

Survey respondents identified several additional data sources for healthy retail research in the open-ended section portion of the survey. See Table 2 for a full list of data sources identified through the convening and follow-up survey.

### 3.4. Research Agenda Findings

Based on the information gathered in steps 2–5 (see Figure 1), a total of 37 research questions, grouped into ten key issue areas, emerged as priorities for future research (Table 3). Five of these issue areas focus on understanding the current food retail environment and consumer behavior and five focus on assessing implementation and effectiveness of interventions and policies to attain healthier retail. 

Through small and large group discussions at the in-person convening, several cross-cutting considerations for future research emerged and were grouped into three themes: potential research partners, data sources, and study designs and settings (Table 4).

## 4. Discussion

This article is the first to present a national agenda for research to support healthy food retail, developed iteratively and collaboratively by experts in public health research, advocacy, and food retail and marketing. This research agenda builds on the 2011 *Harnessing the Power of Supermarkets to Help Reverse Childhood Obesity* report, which proposed in-store marketing strategies developed collaboratively by retailers, researchers, manufacturers, and marketing professionals to encourage the purchase of healthy products while maintaining or improving retailers’ bottom lines [11]. This research agenda reflects advancements in research that have occurred in the intervening years and outlines key areas for future research.

Thirty-seven key research questions, grouped into ten overarching themes, were identified. Priority topics include how frequency, duration, and impact of retailer promotion practices differ by community characteristics and how to leverage federal nutrition assistance programs to support healthy eating. Many of the research questions that received the highest scores in the follow-up survey for importance or health equity received low scores for feasibility, underscoring the need to address barriers to evaluation. Identified strategies to address these barriers include partnerships with retailers, government agencies, business schools, advocacy organizations, and others to implement and evaluate pilot programs and policies, as well as exploration of new study designs and data sharing opportunities. 

Of the ten key research themes that emerged, half centered around describing the current food retail environment and how environmental factors shape consumer behavior. Considering that an estimated three-quarters of purchase decisions are made while shopping, a nuanced understanding of marketing strategies used by manufacturers and retailers and how those strategies drive behavior can guide targeted interventions [23]. Additionally, most research to-date has focused on grocery stores, but changes in the food retail environment, including growth in online retail and proliferation of dollar-stores in low-income and rural areas, point to a need for research on nontraditional retail outlets [24,25,26]. 

The other five key research themes focused on evaluating interventions designed to improve the retail environment and access to nutritious food. The commissioned reviews highlighted evidence of retailer-, researcher- and government-initiated interventions that have led to increased healthy purchases, including fresh fruit and vegetable prescriptions, revisions to the WIC packages, and financial incentives for healthy purchases using SNAP [19,27,28]. Yet, additional research is warranted to evaluate these interventions at a larger scale, in other settings, and over longer periods of time. Evaluation of novel policies through natural experimentation at the state and local level is also needed. As one step toward facilitating such policy evaluation, federal agencies should provide states greater flexibility to innovate. For example, the USDA could approve state or local waiver applications to remove SSBs from eligible SNAP purchases [29]. Considering SNAP serves as an important source of revenue for many retailers, changes in SNAP and other federal nutrition assistance programs could shift the broader food landscape [30].

### 4.1. Implications for Research and Practice

The agenda-setting process centered around promoting health equity, and the research questions identified account for and aim to address health disparities. As researchers and practitioners pursue the policy, systems, and environmental change strategies identified in this agenda, the Equity-Oriented Obesity Prevention Framework developed by Kumanyika can serve as a guide to ensure equity issues continue to be prioritized [31]. Specifically, Kumanyika calls for designing and evaluating interventions using an explicit equity lens that acknowledges the realities of social inequities, incorporates a “people perspective”, and prioritizes community engagement. 

This research agenda can serve as a resource for researchers writing grant applications, retailers seeking to conduct healthy retail pilots on their own or with researchers or advocates, funders drafting requests for proposals, and advocates engaging in organizational strategic planning. In particular, private foundations and federal agencies including the USDA, CDC, and the National Institutes of Health (NIH) should integrate the research themes outlined in this agenda into their strategic plans, ongoing initiatives, and funding priorities. 

While federal agencies have made progress toward recognizing the importance of the food environment and healthy retail as a strategy to reduce disease and disparities, much work remains. For example, in the *National Nutrition Research Roadmap for 2016‒2021*, the federal Interagency Committee on Human Nutrition Research, which includes representatives from USDA and the U.S. Department of Health and Human Services, identified research on food retail as an area of interest [32]. The CDC has acknowledged the importance of the food retail environment in multiple reports and, in 2015, published *Healthier Food Retail: An Action Guide for Public Health Practitioners* [33,34]. The CDC also promotes healthier retail among small, independent retailers through cooperative purchasing initiatives and communities of practice in the High Obesity Program and Racial and Ethnic Approaches to Community Health program. The NIH, between 1975 and 2018, funded more than 200 grants related to healthy food retail, and the *2020–2030 Strategic Plan for NIH Nutrition Research* recognized the important role of the food environment in shaping dietary behavior [35,36]. At the same time, the *Strategic Plan for NIH Obesity Research* only briefly mentions the food environment and does not mention retail [37]. Similarly, healthy retail is missing from the *USDA Science Blueprint* [38].

As federal departments and agencies use this research agenda to guide future funding priorities, coordination and harmonization across these entities are needed to ensure existing efforts are leveraged and amplified and that critical areas are not overlooked. Drawing on recent recommendations from Fleischhacker et al., creation of a new authority for cross-governmental coordination of nutrition research and policy, as well as strengthened authority, coordination, and investment for nutrition research within the NIH and USDA could help to catalyze new science and partnerships [39].

Research on healthy retail requires collaboration across sectors and disciplines, including relationship-building and data sharing between researchers and retailers. Research institutions and funders should provide financial and technical support to advance these efforts without expectation of immediate research deliverables. For example, to improve accessibility and affordability of data, foundations could serve as a conduit between researchers and industry, following the model of the RWJF Health Data for Action program [22]. Another potential model is the Johns Hopkins Bloomberg American Health Initiative, which provides funding to researchers engaged in consultancies and special projects that facilitate cross-sector partnerships [40].

Progress toward meeting the research goals outlined herein should be monitored. In five years, key stakeholders should be re-convened to discuss achievements and remaining gaps. In the intervening years, researchers, retailers, manufacturers, funders, and advocates should convene periodically to foster partnerships and data sharing.

### 4.2. Strengths and Limitations

This study has limitations. First, the list of attendees for the in-person convening was developed with the aim of bringing together groups across research and practice with a mutual interest in promoting health; thus, some interested parties such as manufacturers and trade associations may have been excluded, and the research questions and other ideas generated at the convening may be subject to bias. Additionally, 43 percent of meeting participants did not complete the follow-up survey; therefore, survey results may be impacted by self-selection bias. Finally, the food retail landscape is rapidly evolving, and this agenda reflects priorities identified at a specific period in time. For example, research questions were generated at an in-person convening in January 2020, before widespread awareness of COVID-19 in the U.S. The pandemic brought about important changes in how people in the U.S. purchase groceries and inspired new research questions (e.g., what are the impacts of increased online grocery purchasing; increased at-home food preparation; expansion of the SNAP Online Purchasing Pilot Program?) [21,41].

The methods used in this study, however, are strong. This study used a multi-step, iterative approach to develop the final research agenda. A range of stakeholders who represented diverse disciplines and organizations, including retailers, were engaged in this process. Finally, a focus on health equity was incorporated in every stage of the retail research agenda-setting process, increasing the likelihood that the research questions identified as part of this process will help address disparities in health.

## 5. Conclusions

The food retail environment presents an ideal setting for intervention to improve diet quality and reduce the prevalence of chronic disease and health disparities. The collaborative agenda-setting process, which included representatives from academic, government, advocacy, funding organizations, and industry, built consensus around key research gaps. The research questions identified through this process aim to inform policies and corporate practices that improve the food retail environment, and, ultimately, public health. This agenda can serve as a guide for researchers, funders, and advocates, ensuring that future work fills critical knowledge gaps, promotes equity, and advances policy and practice.

## Figures and Tables

**Table 1:** Definitions of domains used to rank healthy retail research questions in a follow-up survey sent to experts who previously participated in an in-person healthy retail research convening (n = 46).

Term	Definition
Feasibility	What is the likelihood that this research can be conducted successfully and produce valid and reliable results?
Importance	How important is this research to help inform policy, programs, or retailer practice, given the state of the current evidence?
Health equity	How impactful might the results of this research be in ensuring that all people have a fair and just opportunity to be as healthy as possible?

**Table 2:** Data sources for healthy retail research identified at the in-person convening and through the follow-up survey.

Data Source	Accessibility
Store visitor data using cell phone geolocation information from companies such as SafeGraph	Fee
Sales and customer demographic data from companies such as Nielsen and Information Resources Inc. (IRI)	Fee
Sales and loyalty card data from independent or chain retailers	Through partnerships
Prepared food purchase data from university cafeterias	Through partnerships
State electronic benefit transfer redemption data	Through partnerships
Farmers market sales and customer demographic data through the Farmers Register Portal	Free, coming soon
Data collected by federal agencies Customer Expenditure Survey (Bureau of Labor Statistics) National Household Food Acquisition and Purchase Survey (U.S. Department of Agriculture) National Health and Nutrition Examination Survey (Centers for Disease Control and Prevention)	Free, public use, and restricted datasets

**Table 3:** National research agenda questions.

Key Issue Area	Research Question
Understanding the Current Food Retail Environment and Consumer Behavior	
Understanding and describing the retail food marketing environment	How does the healthfulness of foods and beverages available in retail outlets differ by retail format?
	How does the healthfulness of foods and beverages promoted in retail outlets differ by retail format?
	What are the effects of manufacturer trade promotion practices on retailer practices?
	How do frequency and duration of retailer promotions differ by: community characteristics (e.g., race/ethnicity, socioeconomic status)? time of month (e.g., when SNAP benefits are issued)? product characteristics (e.g., healthfulness, category)? retail format (e.g., supermarkets vs. convenience stores)? retail ordering platform (e.g., brick-and-mortar vs. online)? geography (e.g., urban vs. rural)?
Understanding consumer shopping behavior	Which factors influence consumer decision-making at the point of purchase?
	Which factors influence where consumers shop (e.g., shopping at a dollar store vs. supermarket)?
Impact of retailer marketing strategies	What are the impacts of retailer marketing strategies on: consumer behaviors (e.g., purchasing, impulse buying, stockpiling)? consumer health (e.g., diet quality, body mass index, overweight/obesity)? outcomes of importance to retailers (e.g., sales, profitability, brand loyalty)?
	How do the impacts of retailer marketing strategies differ by: consumer characteristics (e.g., race/ethnicity, socioeconomic status, participation in federal nutrition programs)? time of month (e.g., when SNAP benefits are issued)? product characteristics (e.g., healthfulness, category)? geography (e.g., urban vs. rural)?
Understanding targeted food marketing	To what extent do retailers create targeted promotions based on customer characteristics (e.g., race/ethnicity, socioeconomic status, participation in federal nutrition programs)?
	Which food or beverage manufacturers and food categories have deceptive marketing or front-of-package claims?
Role of emerging retail formats in supporting healthy food access	How do dollar stores affect a community’s access to healthful food?
**Implementation and Effectiveness of Interventions and Policies to Attain Healthier Retail**	
Supporting healthy purchases and reducing unhealthy purchases	What is the optimal design of a retail environment to support healthy eating?
	What changes to retailer marketing strategies improve the healthfulness of food purchases?
	What changes to product packaging, labeling, and/or portion size improve the healthfulness of food purchases?
	What are effective digital strategies to improve the healthfulness of food purchases?
Leveraging SNAP to support healthy eating	What is the impact of increasing the SNAP benefit amount?
	What is the impact of changing the frequency and/or timing of SNAP distribution (e.g., benefits issued twice per month or benefits issued on different days of the month)?
	What is the impact of changing the list of products eligible for purchase with SNAP (e.g., sugar-sweetened beverages)?
	What is the impact of offering produce boxes to SNAP beneficiaries?
	What is the impact of providing incentives for healthy foods for SNAP beneficiaries (e.g., discounts or matching dollars for purchases of whole grains, fruits and vegetables)?
Limiting unhealthy food establishments	How do zoning restrictions for unhealthy food retailers impact access to healthy food in the community?
Addressing social determinants of health	How do interventions or policies that address social determinants of health (e.g., universal basic income, increased minimum wage) impact food and beverage purchasing and consumption?
Assessing differential impacts	How do the impacts of interventions and policies differ by: consumer characteristics (e.g., race/ethnicity, socioeconomic status, participation in federal nutrition programs)? product characteristics (e.g., healthfulness, category)? retail format (e.g., supermarkets vs. convenience stores)? retail ordering platform (e.g., brick-and-mortar vs. online)? geography (e.g., urban vs. rural)?

**Table 4:** Cross-cutting considerations for future research on healthy food retail discussed by in-person convening participants.

Theme	Consideration
Research partnerships	Build long-term relationships with retailers and manufacturers to facilitate the implementation and evaluation of in-store interventions and access to proprietary data
	Collaborate with nontraditional partners, including trade associations, growers and distributors, marketing firms, business schools, advocacy groups, and retailers connected to academic research institutions (e.g., university hospitals, cafeterias, campus stores)
Data sources	Increase access to federal data sources (e.g., SNAP redemption data)
	Make data accessible and affordable to researchers through programs modeled after RWJF Health Data for Action, which serves as a conduit between data owners and researchers [22]
Study design and setting	Study nontraditional retailers, including supercenters, dollar stores, and online retailers
	Use a variety of study designs (e.g., laboratory experiments, pilot programs, randomized controlled trials, longitudinal evaluations)
	Draw lessons from interventions or policies abroad
	Promote innovative data collection approaches, such as investigative journalism or federally or congressionally commissioned investigations

## Appendix A

**Table A1:** Follow-up survey participant (n = 26) mean rankings of research questions in terms of feasibility, equity, and importance.

Key Issue Area	Research Question	Feasibility	Equity	Importance	Composite
Understanding the Current Food Retail Environment and Consumer Behavior					
Understanding and describing the retail food marketing environment	How does the healthfulness of foods and beverages available in retail outlets differ by retail format?	4.35	3.62	3.35	3.77
	How does the healthfulness of foods and beverages promoted in retail outlets differ by retail format?	4.12	4.00	3.81	3.97
	What are the effects of manufacturer trade promotion practices on retailer practices?	2.52	3.60	3.88	3.36
	How important is revenue from trade promotion to retailers? (e.g., what proportion of total revenue comes from trade promotion?) *	2.26	3.04	3.44	2.93
	How do frequency and duration of retailer promotions differ by…				
	community characteristics (e.g., race/ethnicity, socioeconomic status)?	3.77	4.50	4.15	4.14
	time of month (e.g., when SNAP benefits are issued)?	3.65	4.27	4.08	4.00
	product characteristics (e.g., healthfulness, category)?	3.92	3.40	3.36	3.56
	retail format (e.g., supermarkets vs. convenience stores)?	3.72	3.36	3.08	3.39
	retail ordering platform (e.g., brick-and-mortar vs. online)?	3.48	3.31	3.50	3.43
	geography (e.g., urban vs. rural)?	3.88	3.85	3.54	3.76
Understanding consumer shopping behavior	Which factors influence consumer decision-making at the point of purchase?	4.04	3.62	3.73	3.79
	Which factors influence where consumers shop (e.g., shopping at a dollar store vs. supermarket)?	4.00	3.81	3.46	3.76
Impact of retailer marketing strategies	What are the impacts of retailer marketing strategies on…				
	consumer behaviors (e.g., purchasing, impulse buying, stockpiling)?	3.50	3.69	4.04	3.74
	consumer health (e.g., diet quality, body mass index, overweight/obesity)?	2.54	3.88	4.31	3.58
	outcomes of importance to retailers (e.g., sales, profitability, brand loyalty)?	2.92	2.85	3.80	3.18
	How do the impacts of retailer marketing strategies differ by…				
	consumer characteristics (e.g., race/ethnicity, socioeconomic status, participation in federal nutrition programs)?	3.46	4.52	4.40	4.14
	time of month (e.g., when SNAP benefits are issued)?	3.52	4.12	4.12	3.92
	product characteristics (e.g., healthfulness, category)?	3.60	3.20	3.36	3.39
	retail format (e.g., supermarkets vs. convenience stores)? *	3.40	3.08	2.96	3.15
	retail ordering platform (e.g., brick-and-mortar vs. online)? *	3.29	2.84	3.40	3.18
	geography (e.g., urban vs. rural)?	3.22	3.70	3.48	3.46
Understanding targeted food marketing	To what extent do retailers create targeted promotions based on customer characteristics (e.g., race/ethnicity, socioeconomic status, participation in federal nutrition programs)?	3.00	4.48	4.52	4.01
	Which food or beverage manufacturers and food categories have deceptive marketing or front-of-package claims?	4.00	3.32	3.52	3.61
Role of emerging retail formats in supporting healthy food access	How do dollar stores affect a community’s access to healthful food?	3.64	4.31	4.08	4.01
**Implementation and Effectiveness of Interventions and Policies to Attain Healthier Retail**					
Supporting healthy purchases and reducing unhealthy purchases	What is the optimal design of a retail environment to support healthy eating?	2.74	3.74	4.30	3.59
	What changes to retailer marketing strategies improve the healthfulness of food purchases?	3.09	3.91	4.22	3.74
	What changes to product packaging, labeling, and/or portion size improve the healthfulness of food purchases?	3.26	3.52	3.78	3.52
	What are effective digital strategies to improve the healthfulness of food purchases?	3.61	3.61	3.91	3.71
Leveraging SNAP to support healthy eating	What is the impact of increasing the SNAP benefit amount?	3.70	4.74	4.52	4.32
	What is the impact of changing the frequency and/or timing of SNAP distribution (e.g., benefits issued twice per month or benefits issued on different days of the month)?	3.35	4.17	3.83	3.78
	What is the impact of changing the list of products eligible for purchase with SNAP (e.g., sugar-sweetened beverages)?	3.30	4.22	4.35	3.96
	What is the impact of offering produce boxes to SNAP beneficiaries?	3.48	3.74	3.43	3.55
	What is the impact of providing incentives for healthy foods for SNAP beneficiaries (e.g., discounts or matching dollars for purchases of whole grains, fruits and vegetables)?	3.96	4.43	4.09	4.16
Limiting unhealthy food establishments	How do zoning restrictions for unhealthy food retailers impact access to healthy food in the community?	2.61	3.83	3.70	3.38
Addressing social determinants of health	How do interventions or policies that address social determinants of health (e.g., universal basic income, increased minimum wage) impact food and beverage purchasing and consumption?	2.52	4.73	4.59	3.93
Assessing differential impacts	How do the impacts of interventions and policies differ by…				
	consumer characteristics (e.g., race/ethnicity, socioeconomic status, participation in federal nutrition programs)?	3.65	4.30	4.30	4.09
	product characteristics (e.g., healthfulness, category)?	3.70	3.00	3.26	3.32
	retail format (e.g., supermarkets vs. convenience stores)?	3.78	3.26	3.30	3.45
	retail ordering platform (e.g., brick-and-mortar vs. online)?	3.36	3.26	3.57	3.40
	geography (e.g., urban vs. rural)?	3.70	4.00	3.78	3.83

* Indicates question was eliminated due to low composite score or low score for equity or importance (<3).
